# Quantifying the medical student learning curve for ECG rhythm strip interpretation using deliberate practice

**DOI:** 10.3205/zma001248

**Published:** 2019-08-15

**Authors:** Jason Waechter, David Reading, Chel Hee Lee, Mathieu Walker

**Affiliations:** 1 University of Calgary, Depts. of Critical Care and Anesthesiology, Calgary (Alberta), Canada; 2 University of British Columbia, Dept. of Internal Medicine, Vancouver (British Columbia), Canada; 3 University of Calgary, Dept. of Mathematics and Statistics and Dept. of Critical Care, Calgary (Alberta), Canada; 4 University of McGill, Dept. of Medicine, Division of Cardiology, Montreal (Quebec), Canada

**Keywords:** learning curve, Electrocardiography, Competency-Based Education, deliberate practice

## Abstract

**Objectives: **Obtaining competency in medical skills such as interpretation of electrocardiograms (ECGs) requires repeated practice and feedback. Structured repeated practice and feedback for ECGs is likely not provided to most medical students, so skill development is dependent on opportunistic training during clinical rotations. Our aim was to describe:

1. the amount of deliberate practice completed for learning ECG rhythm strip diagnoses in first year medical students,
2. the learning curve for rhythm strip diagnosis, and
3. student experiences with deliberate practice.

**Methods: **First year medical students from two medical schools were provided with online rhythm strip practice cases. Diagnostic accuracy was measured throughout practice, and students were provided feedback for every case they completed. Total cases practiced and time spent practicing were correlated with their performance during practice and on an exam.

**Results: **314 of 384 (82%) students consented. The mean number of ECGs each student practiced was 59 (range 0-280), representing 18,466 total instances of deliberate practice. We generated mathematical models that accurately correlated both the number of cases practiced and time spent practicing, with diagnostic accuracy on an exam (p<0.001). For example, students would need to spend on average of 112 minutes and complete 34 practice cases to obtain 75% on an ECG rhythm strip exam. Student satisfaction was high using the online cases.

**Conclusions: **We succeeded in delivering deliberate practice for ECG rhythm strip interpretation to a large cohort of students at 2 medical schools. We quantified a learning curve that estimates the number of cases and practice time required to achieve pre-determined levels of diagnostic accuracy. This data can help inform a competency-based approach to curriculum development.

## Zusammenfassung

**Ziele: **Der Kompetenzerwerb von medizinischen Fähigkeiten wie der Interpretation von Elektrokardiogrammen (EKGs) erfordert wiederholtes Üben und Feedback. Strukturierte wiederholte Übungen und Feedback für die Durchführung von EKGs stehen den meisten Medizinstudierenden in der Regel nicht zur Verfügung, sodass der Kompetenzerwerb von einer opportunistischen Ausbildung im Rahmen klinischer Praktika abhängt. Unser Ziel war die Untersuchung folgender Aspekte:

1. die Anzahl der bewussten Übungen zum Erlernen der EKG-Rhythmusstreifendiagnose bei Medizinstudierenden im ersten Jahr,
2. die Lernkurve für eine Rhythmusstreifendiagnose und
3. die Erfahrungen der Studierenden mit bewusstem Üben.

**Methodik: **Medizinstudierenden des ersten Jahrgangs zweier medizinischer Fakultäten wurden online Übungsfälle zum Thema Rhythmusstreifen bereitgestellt. Die diagnostische Genauigkeit wurde während der gesamten Übungen gemessen und die Studierenden erhielten für jeden abgeschlossenen Fall ein Feedback. Die Gesamtzahl der geübten Fälle und die Zeit, die mit dem Üben verbracht wurde, wurden mit ihrer Leistung während der Übungen und bei einer Prüfung korreliert.

**Resultate: **314 von 384 (82%) Studierende willigten ein. Die durchschnittliche Anzahl der EKGs, mit denen jeder Studierende übte, betrug 59 (Gesamtanzahl 0-280), was einer Gesamtzahl von 18.466 Fällen bewusster Praxis entspricht. Wir generierten mathematische Modelle, die sowohl die Anzahl der geübten Fälle als auch die Zeit, die mit der Übung verbracht wurde, genau mit der diagnostischen Korrektheit bei einer Prüfung korrelierten (p<0.001). Beispielsweise mussten die Studierenden durchschnittlich 112 Minuten und 34 Übungsfälle absolvieren, um 75% für eine EKG-Rhythmusstreifenprüfung zu erhalten. Die Zufriedenheit der Studierenden war bei den Online-Fällen hoch.

**Schlussfolgerungen: **Es ist uns gelungen, einer großen Gruppe von Studierenden an zwei medizinischen Fakultäten eine gezielte Übung zur Interpretation von EKG-Rhythmusstreifen bereitzustellen. Wir quantifizierten eine Lernkurve, welche eine Schätzung der Anzahl der Fälle und die Übungszeit darstellt, die benötigt werden, um ein vorgegebenes, diagnostisches Genauigkeitsniveau zu erreichen. Diese Daten können zur Entwicklung eines kompetenzbasierten Curriculum-Ansatzes beitragen.

## Introduction

Although electrocardiogram (ECG) interpretation is a core competency for all graduating medical students [1], literature from the past 3 decades has consistently shown that ECG interpretation skills are consistently below expectations for graduating medical students [2], [3], residents in training [4], [5], [6], [7], [8], [9], and physicians in practice [10], [11], [12]. ECG interpretation errors may expose patients to harm through delayed diagnosis, inappropriate investigations and treatment, and delays in appropriate treatment [13], [14]. There is an identified need for improvement to ECG learning in both undergraduate and residency training programs [15].

Unfortunately, variability and low performance of medical skills is not limited to ECG interpretation. High variability of procedural competence among residents, fellows, and attendings has been described for lumbar puncture [16], bariatric surgery [17], and insertion of intravascular catheters [18]. Such outcomes have generated calls for prompt changes to medical education [19].

Deliberate practice is widely cited as a key component of obtaining competence and mastery [20], [21]. Deliberate practice, a process of providing multiple iterations of structured practice and feedback until the trainee demonstrates the required competency [19], [22], [23], [24], has been described as superior to passive learning for skills acquisition [25], [26], [27]. Deliberate practice to obtain mastery has been demonstrated in multiple domains including sports, gaming, the business world, and musical performance [28], [29]. Within medicine, studies have shown improved performance of specific skills via mastery learning approaches, including paracentesis, central line placement, and advanced cardiac life support skills [18], [30], [31].

The Clerkship Directors of Internal Medicine (CDIM) survey results in 2005 suggested that the majority of time spent on ECG teaching during clerkship was focused on didactic theory, with little formalized structured ECG practice [32]. Indeed, limited opportunities for practice and feedback have been identified as contributing to poor ECG interpretation skills [1].

The premise of a competency based learning approach is promotion based on performance that meets established standards. A population of students will demonstrate different learning rates for a given skill and will require different amounts of practice and feedback to obtain competency [32], [33]. Therefore, a flexible delivery of practice and feedback should help individualize training for each student’s needs. Further, the optimal number of ECG cases that need to be practiced to achieve competence, and the time required to complete this practice and feedback would be helpful in planning resources for learning ECGs; this is currently unknown [34].

The first objective of this paper was to describe the amount of deliberate practice completed by a cohort of first year medical students who were learning ECG rhythm strip diagnoses. Second, we aimed to mathematically quantify the learning curve for rhythm strip diagnosis with respect to both time and number of cases practiced. The third objective was to describe student opinions regarding their experiences with deliberate practice.

## Methods

This was an observational associational cohort study that used a novel online platform to measure and describe practice behaviours and performance of medical students learning ECG rhythm strip diagnoses. An observational design was chosen because an experimental design was not possible for reasons of disparate treatment between intervention and control groups due to the absence of an appropriate pre-existing intervention with which to compare [35].

### Setting and participants

A convenience sample of first year medical students enrolled at 2 medical schools in Canada (McGill University and University of Calgary) in 2016 and 2017 were invited to participate in the study during their Cardiovascular courses. McGill offers 7 hours of lecture and 2 hours of workshop on ECG learning in a 6 week course; Calgary provides 7 hours of lecture and 0 hours of workshop in a 12 week course that is combined with the Respiratory curriculum. Both curricula include case based learning and some of these cases incorporate ECG’s. Students were invited to participate via e-mail sent by the undergraduate office and in-class announcements. The University of Calgary Conjoint Health Research Ethics Board approved the study. No funding was obtained for this study.

Students practiced rhythm strip cases through the freely accessible online modules on [https://www.teachingmedicine.com/] during independent study time. The 14 different ECG diagnoses chosen for learning were based on the Advanced Cardiac Life Support (ACLS) rhythms that must be mastered in order to manage a cardiac arrest (see table 1 (Tab. 1)). The ECG’s were presented as rhythm strips (as opposed to 12 lead ECGs). There were 3 discrete practice modules, each with one different example of the 14 diagnoses (including one normal rhythm). Thus, completion of one module guaranteed one exposure to each of the 14 diagnoses. Students were required to complete at least one practice module as part of their coursework. Students could practice each module as many times as they wanted. Performance during practice was quantified as the percentage of rhythm strips that were diagnosed correctly by each student, as compared to the correct answer that was unanimously agreed upon by 3 experts. Time to complete the practice modules was recorded by the online platform. The timer started when the module was entered and stopped when the module was exited. If there was no user activity for greater than 5 minutes, then 4 minutes was subtracted and the timer was stopped.

Each rhythm strip case required the student to answer 8 questions characterizing the ECG, followed by 2 questions about the diagnosis (see table 2 (Tab. 2)). Except for the question on ventricular rate, all questions were multiple-choice with the diagnosis question providing 14 options. Motivation techniques, such as showing students their evolving performance, popup messages that rewarded and encouraged excellent performance, and providing comparison statistics between the user and the mean scores of their peers were built into the practice modules in an attempt to increase student engagement [36].

Multiple considerations informed the design of the feedback that was provided [37]. Four types of immediate feedback were available to each student. First, answers were identified as correct or incorrect. Second, if the submitted diagnosis was incorrect, a table displayed the correct diagnosis, the student’s incorrect diagnosis, the diagnostic criteria of both diagnoses with highlighted differences between the two sets of criteria. Third, an explanatory discussion of the case was provided. The fourth form of feedback was the opportunity to ask questions of the course instructor via email, with an email response provided within 24 hours. 

All student responses were recorded and made available for analysis. At the end of their Cardiovascular course, students completed a mandatory rhythm strip exam that counted toward 5% of their final course grade. Exam results from McGill were excluded from analysis because the exam questions were taken from the practice modules and thus did not represent a valid examination of previously unseen cases. The University of Calgary used an offline paper exam which contained no rhythm strips from the practice modules. Each rhythm strip exam case was comprised of a single diagnosis that was considered by 2 experts to be similar in difficulty to the practice cases, and contained the same diagnoses as the rhythm strips in the practice modules. Performance on the exam was defined as the percentage of rhythm strips that were correctly diagnosed. Failure to complete the mandatory practice module or the exam resulted in an “Incomplete” status in the course. 

#### Data analysis

Data was de-identified and exported to a local computer. Data was analyzed using R-3.5.1 [38], nlme-3.1-137 [39], Stata 10.0 (Statacorp LLP) and Excel software (Microsoft Excel for Mac, Version 15.28). Descriptive statistics, including means (with standard deviations [SD]) and proportions, were used to summarize survey data. 

#### Practice data

This data is comprised of two independent populations, is non-linear and compares multiple paired samples per individual; the individuals do not all have the same number of paired samples. Two scatter plots were created to observe the relationships between diagnostic accuracy and time per module spent practicing vs. the number of modules completed. The Gompertz function was used to graphically represent the means of this data [40]. Differences between the 2 schools were assessed using ANCOVA. Differences between paired successive modules were tested for significance using paired t-tests with Bonferoni correction.

#### Exam data

This data is comprised of one population, is non-linear, unpaired and it is assumed that all observations are independently measured. One scatter plot was created to observe the relationship between diagnostic accuracy and total time spent practicing vs. the number of modules completed. Based on visual inspection of this scatter plot, three mathematical functions: Gompertz function, Michaelis-Menten (MM) function, and quadratic function were chosen as possible candidates to represent these 2 relationships [40]. The coefficients associated with the models were assessed for statistical significance of their fit to the data. Alpha was set to 0.05 a priori. Akaike information criteria (AIC) and correlation between observed and predicted measurements are used for measuring the goodness fit of the mathematical models. 

## Results

### Amount of practice

**Figure 1:**
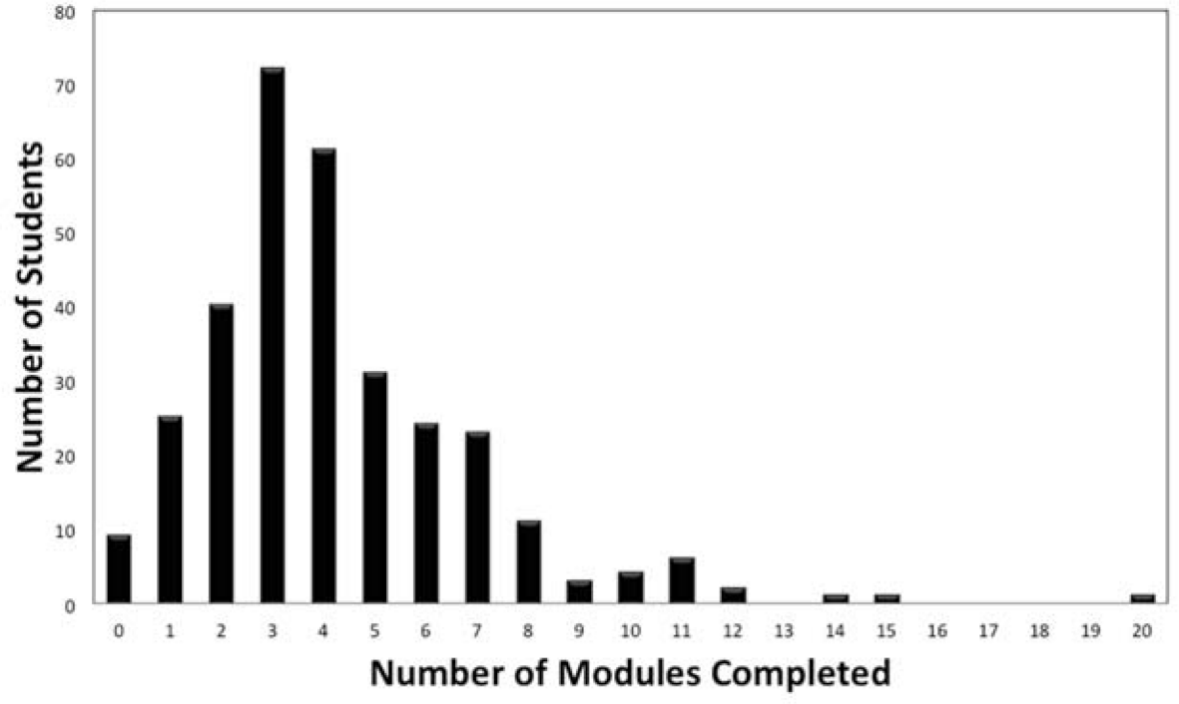
The number of modules completed by each student. One module contains 14 rhythm strip cases.

A total of 384 students were invited to participate and 314 (82%) consented. Among the 298 (95%) of consented participants who completed the pre-survey, the mean age was 24.9 (SD=4.2) years and 54% were female. 14 (4%) students indicated they had previously used online practice modules for rhythm strip learning. The mean number of times each of the 14 diagnoses were practiced per student was 4.1 (SD=2.7), representing 59 rhythm strips practiced per student (see figure 1 (Fig. 1)). A total of 24 (8%) students practiced only the mandatory single module prior to the exam. Nine students did not complete the mandatory module prior to the exam and completed it after the exam. Forty-eight students completed 10 or more modules, which translated to ≥140 rhythm strips each. The maximum number of modules completed by a student was 20, representing 280 rhythm strips. The total number of rhythm strips practiced by all 314 students was 18,466 and thus 18,466 instances of feedback were delivered.

#### Practice performance

**Figure 2:**
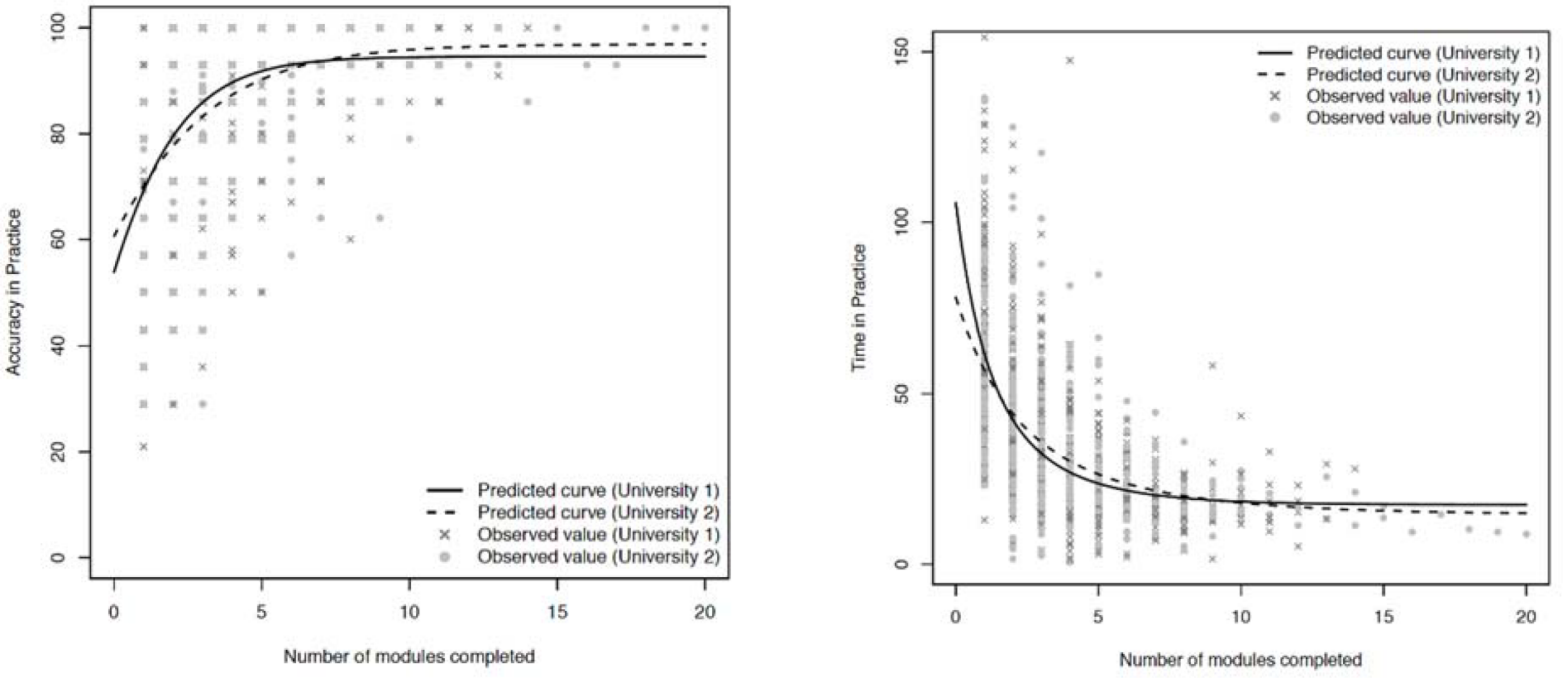
Left shows diagnostic accuracy during practice of two difference medical schools during rhythm strip practice. Right shows per module practice time. There are no significant differences between the two schools except for one time difference at module #3, which is likely not “clinically” significant.

Figure 2 (Fig. 2), left and right shows the scatter plots relating the number of practice modules completed with diagnostic accuracy and per module practice time in two different schools during practice. There were no statistical differences in diagnostic accuracy during practice between the two schools. There were no statistical differences in per module practice time between the two schools except for one time point (at module 3). Differences in diagnostic accuracy between consecutive modules showed statistically significant increases between modules: 1 and 2; 2 and 3; not 3 and 4, but again 4 and 5, indicating that diagnostic accuracy was continuing to increase through modules 1-3 and possibly also continuing up to module 5 (full statistical values reported in online supplemental material Nr. 1 see attachment 1 ). Differences in per module practice time significantly decreased between consecutive modules: 1 and 2; through to modules 7 and 8 inclusive, indicating that speed was continuing to increase throughout all these modules.

#### Exam performance

**Figure 3:**
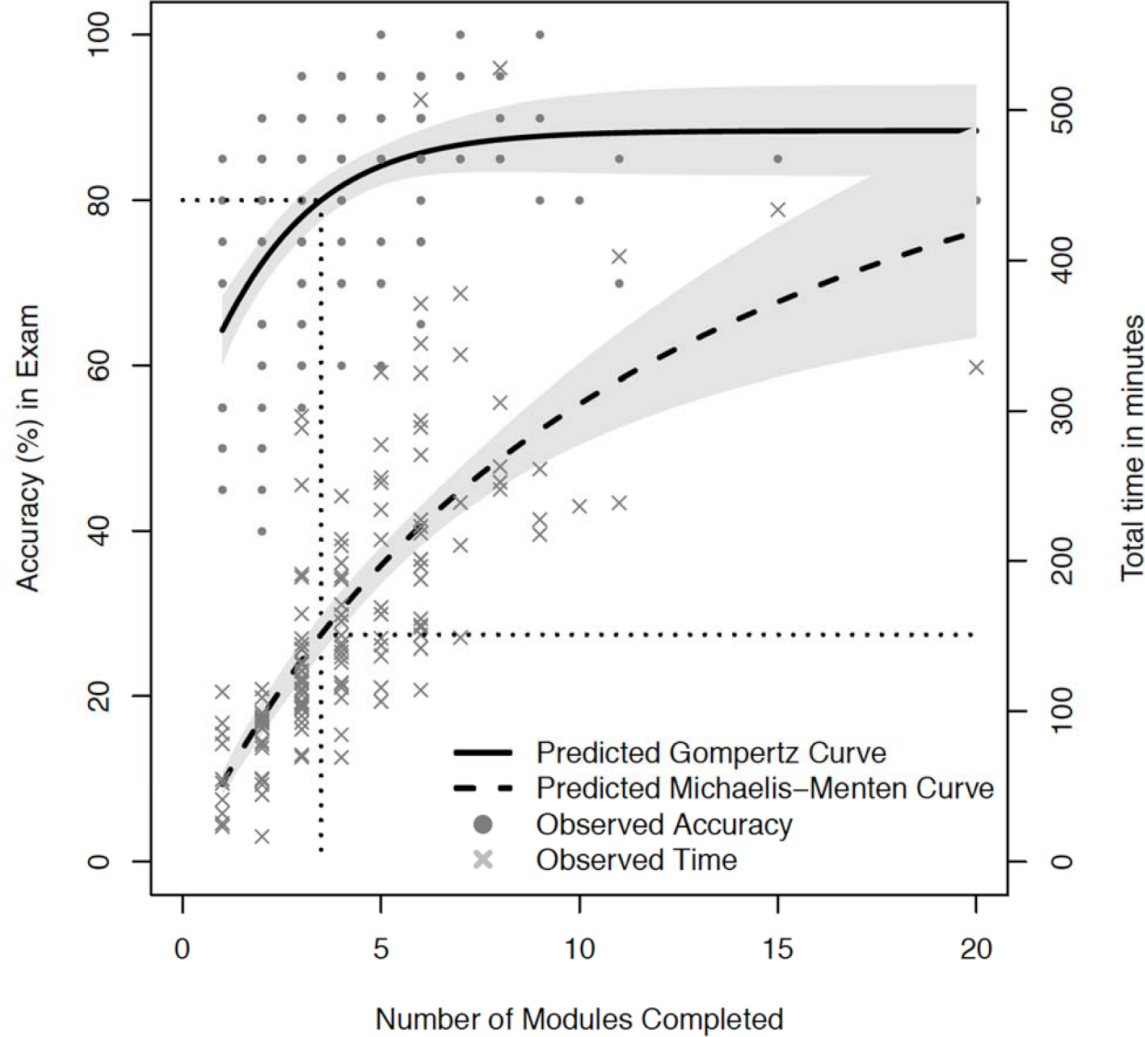
Diagnostic accuracy and total practice time are plotted against the total number of modules practiced. The solid line curve is the Gompertz curve that estimates diagnostic accuracy. The dashed curve is the Michaelis-Menten curve that estimates total practice time. Grey shaded regions represent 95% CI. The horizontal and vertical dotted line shows an exemplar 80% diagnostic accuracy mapping to the estimated number of modules and total practice time required to obtain this competency level. See also tab. 3 for calculated values.

The mathematical function that best approximated the diagnostic accuracy learning curve was the Gompertz equation with the following coefficients: Y~88*exp (0.51*exp (0.47*M)). The function that best described the total practice time curve was the Michaelis-Menten function with the coefficients: Y~671*M/(12.1+M). M represents the number of modules completed. The AIC goodness of fit assessment for both of these mathematical functions produced p values<0.001 for all coefficients in the models. These curves are used to estimate the workload required by students to obtain a pre-defined level of performance on the exam. A two-stage model for finding the time spent practicing from the expected accuracy is illustrated in figure 3 (Fig. 3). The estimate Gompertz model is used to find the corresponding required number of modules, which is then used as an input for the estimated Michaelis-Menten function to predict the time needed for practice. Table 3 (Tab. 3) shows the total practice time and required number of modules practiced to achieve scores ranging from 70 to 85% on the exam. 

In the pre-survey, students reported how much time they spent practicing rhythm strip interpretations prior to the research study; when students were stratified according to pre-study practice, there were no statistical differences in exam performance.

#### Student satisfaction

Based on the survey completed at the end of the Cardiovascular courses, 174 (of 314 who consented) students indicated that the learning modules were effective (97%), efficient (97%) and enjoyable (92%) (online supplement material Nr. 2 see attachment 2 ). Most students believed practice is required to learn ECG interpretation (99%), that immediate feedback was helpful (95%), and that they were interested in using the same method for learning other diagnostic skills, such as x-ray and ultrasound interpretation (99%). 

#### Instructor workload

In terms of instructor workload to respond to feedback emails from the students, a total of 14 email questions were submitted by students from McGill and 37 were submitted by Calgary students. Comparing emails to total cases practiced, 51 feedback emails were submitted for 18,466 practice cases, which is approximately 1 email for every 360 cases practiced.

## Discussion

Our data shows that first year medical students practiced interpreting a mean of 59 rhythm strips prior to a rhythm strip exam and in total, over 18,000 instances of feedback were provided to 314 students at 2 different medical schools. This is important for several reasons. First, challenges have been reported on providing feedback for deliberate practice [37]. We have described the details of successfully providing feedback for deliberate practice to two large cohorts of medical students, thus addressing a need identified within medical education literature.

Second, our previously unpublished local data demonstrated that when practice was not mandated and there was no rhythm strip exam, students completed a mean of only 6 practice rhythm strips despite strong promotion and encouragement to practice by instructors [41]. Comparing these findings to the findings of our current study, introducing a mandatory practice module and a dedicated rhythm strip summative exam resulted in a 9-fold increase in practice behaviours by the students. The students were willing, able and motivated to practice well beyond the mandated workload by the course instructors, completing a mean of 59 practice rhythm strips when only 14 were required.

The dedicated rhythm strip summative exam was likely a strong motivator for student practice behaviour. Other studies have described the positive impact of summative assessments specifically on student performance on ECG interpretation [42], [43]. In residency training programs, periodic objective assessments of ECG interpretation skills have been recommended [34]. In addition to formative feedback, Raupach et al. have identified that summative feedback increased medium term retention of ECG interpretation skills regardless of teaching technique and thus, should be strongly considered as an important component for skill retention [44].

The learning curve for ECG rhythm strip interpretation in medical students has not been previously quantified. A learning curve is a graphic illustration relating a metric of time or effort with performance of a skill [22]. We demonstrated that the diagnostic accuracy and time spent practicing were very similar between students at two different medical schools. Students continued to demonstrate improvement both in diagnostic accuracy and speed of diagnosis while practicing up to 4 modules (56 cases). The value of 56 cases emphasizes the large number of practice cases that should be made available to students so that they have the opportunity to continue to practice while they are continuing to improve. Identifying when the learning curve reaches its asymptote is important to indicate when learning probably stops.

Using the curves in figure 3 (Fig. 3), the number of ECG cases and the time required to obtain pre-determined levels of competency in first year medical students can be determined (see table 3 (Tab. 3)). This information is valuable for students to help them budget their study time and set realistic goals of performance. The results of the learning curve analysis can also provide direction for curriculum development; a competency standard of diagnostic accuracy can first be chosen and the learning curves estimate how much deliberate practice is required for the students. For example, if the competency standard is set to 75% diagnostic accuracy, 34 practice cases and 111 minutes of practice will be required for the average medical student to achieve this level of performance. We feel strongly that the responsibility of providing the required practice and feedback to obtain medical skills belongs to the medical school and its faculty, in contrast to being imposed on the students.

Previous calls for changes to how ECG interpretation is taught suggests that more practice opportunities are required [2]. The 2013 CDIM survey of internal medicine clerkship programs reported on the number of 12 lead ECGs that students in third year clerkship rotations formally interpreted under supervision [1]. Students from only 37% of medical schools formally interpreted more than ten 12-lead ECGs during their internal medicine rotation [1]. All other respondents indicated that their students interpreted fewer ECGs, or did not respond to this question. Data for pre-clerkship ECG training, which is the population we studied, is sparse. 

Our study investigated rhythm strip interpretation and not 12 lead ECG interpretation. However, 12 lead ECG interpretation is more complex than rhythm strips and thus, could be expected to require even more practice than rhythm strips to achieve competency. Our results, combined with the results of the CDIM survey, may suggest that the ECG practice needs of students are not being met at many medical schools. To further highlight the gap between what is provided to trainees and what is needed by trainees, the ACC/AHA guidelines for learning 12 lead ECGs recommend that initial learning should incorporate a minimum of 500 supervised interpretations [45], although the evidence for this recommendation is unclear.

One of the key components of skill development is feedback [46]. Formative feedback can help students self-monitor and determine if they have met the competency standards defined by their school [47], [48]. The quality and nature of feedback has a strong influence on motivation [49], and is suggested to be most useful when instructors assume an active role in the learning process [50]. An absence of feedback or lack of support can lead to low motivation and early termination of self-directed courses [49]. Therefore, a self-directed learning resource should incorporate direct involvement of instructors. Our learning tool provided a blended form of feedback, including 3 different components of automated feedback, and an email function that enabled efficient involvement of the local instructor.

There are logistical challenges to providing individualized feedback to students when there are many students and few instructors. The first consideration is scalability and feasibility. For example, for a class of 100 students where each student completes 50 practice ECGs, feedback would be required 5,000 times. To provide deliberate practice to our cohort of 314 students, 18,466 instances of both practice and feedback were required. With computer algorithms providing automated feedback, the workload of providing additional email feedback was low for the instructors. The workload of additional feedback that was requested from students via email to their local instructors was on average, 25 emails per instructor and not deemed overwhelming. Viewed another way, there was one feedback email question for every 360 cases practiced.

Another advantage of automated feedback is consistency; all learners receive the same quality of feedback because the influence of assessor variability is removed from the process. Additionally, the quality of the feedback can be increased over time as performance data of students is collected and analyzed and common errors are identified. This information informs modifications to case discussions, so that common errors can be directly and pre-emptively addressed.

A positive emotional response to a learning environment facilitates student engagement [51], [52]. Our survey data confirmed that 92% of students described the deliberate practice learning process as “very fun” or “a little enjoyable”. Student experiences may also have been positively influenced by their perception that the learning resources were both effective and efficient. Additionally, individualized feedback likely also contributed to both student satisfaction and performance, given that students indicated that instant feedback was either “absolutely required” (95%) or “helpful” (4%). 

Strengths of this study include unobtrusive collection of learning analytics during core curricular activities, thereby minimizing participant bias and maximizing data collection. Enrolling students from 2 medical schools increases the generalizability of our findings. The study was easy to implement and will be scalable for future study of other diagnostic skills.

There are many limitations to our research. A comparative experimental design is methodologically stronger, but the absence of a control group with whom we could justify equipoise was not possible. No previous method of providing deliberate practice existed at either medical school and we could not randomize students into a group that did not practice; therefore, our study was observational.

Our independent variable was quantified on the basis of module completion, and not individual case completion. If we had structured our data collection on individual cases completed instead of the modules completed, we would have obtained higher fidelity data. Realizing this limitation after data collection, we have modified the data collection software to enable future projects to analyze case by case data.

Post analysis, we realize that our digital practice library is likely too small to meet the needs of the average student should we target a diagnostic accuracy of 80% or greater. Further, dividing the cases into 3 separate modules likely creates artificial and possibly meaningless stop points for the learner; it would probably be better to have all practice cases within one module, and have well in excess of 50 practice cases to help ensure there are enough cases for students at the slower end of the learning curve. As we have a total of 42 unique cases, we fall short of this goal and this is a limitation in our study because students who practiced more than 42 cases were repeating cases they had previously practiced.

We measured short term retention of rhythm strip interpretation; the exact timing of when the students completed practice was not measured, but was contained within a 10 week learning block and it is probable that a lot of practicing occurred within a 2-3 week proximity of the exam. We cannot extrapolate our results to long term retention, but hope to be able to re-assess students in more senior years of training and repeat our analysis with long term (2 years) retention.

We studied performance in ACLS level rhythm strip ECG interpretation and these results cannot be extrapolated to full 12 lead ECG interpretation, which involves more analysis, and often multiple co-existing diagnoses.

Importantly, the students were interpreting the ECG rhythms without any clinical context; diagnostic interpretation might change when patient information is provided [53], [54]. 

To our knowledge, our students used primarily one learning tool for practicing ECG’s. Therefore, we cannot extrapolate our results to other learning tools; it would, however, be very interesting to compare learning curve slopes for different tools so that the tool that produces the fastest learning with least effort could be identified. Further, our assessment tool (the rhythm strip exam) has not yet been formally validated.

Another limitation in our study is the lack of analysis on each individual learner; we are reporting means of performance across many students. Not all learners follow the same learning curve; in fact, the mean of student performance rarely describes the performance of an individual student [55].

In conclusion, we successfully provided deliberate practice to a large cohort of first year medical students and our data quantifies a learning curve for ACLS level rhythm strip interpretation using a specific online learning module. These results may assist in curricular design for ECG rhythm strip interpretation, a required skill for managing cardiac arrest.

## Notes

Jason Waechter is the founder of teachingmedicine.com. The modules used for this project are currently open access and freely accessible.

### Ethical approval

The University of Calgary Conjoint Health Research Ethics Board has approved this research study (REB14-0654_MOD2). The University of McGill has approved this research study (IRB study number A07-E50-15B).

#### Previous presentations

Poster presentation titled “Learning Curves for ECG Interpretation: Correlating Deliberate Practice with Performance.” Presented at the 2017 Canadian Conference for Medical Education (CCME)

## Acknowledgement

We would like to thank Dr. Rachel Ellaway and Dr. Martin Pusic both for editing the manuscript and providing support for the project.

## Competing interests

The authors declare that they have no competing interests. 

## Supplementary Material

Statistical analysis of practice data

Pre-survey completed by 298 students

## Figures and Tables

**Table 1:**
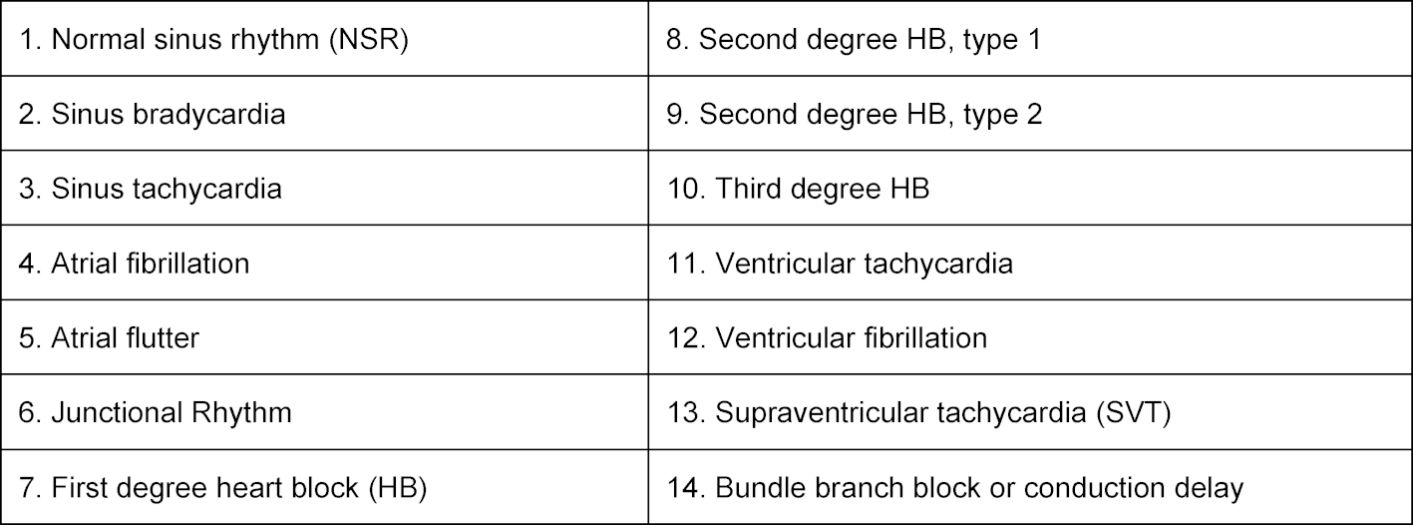
Diagnoses Included in Practice Cases

**Table 2:**
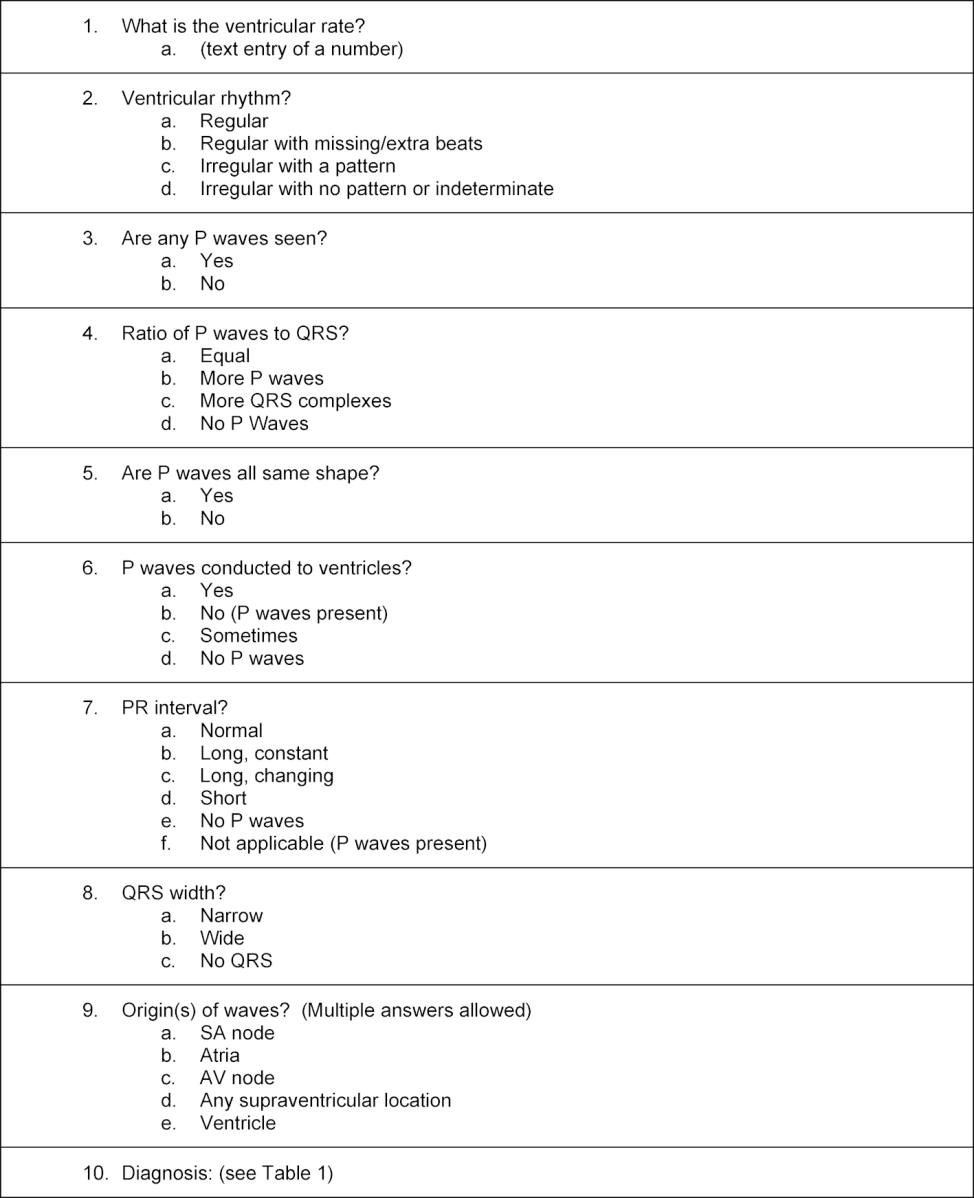
Questions and Possible Answers Asked for Each Rhythm Strip.

**Table 3:**
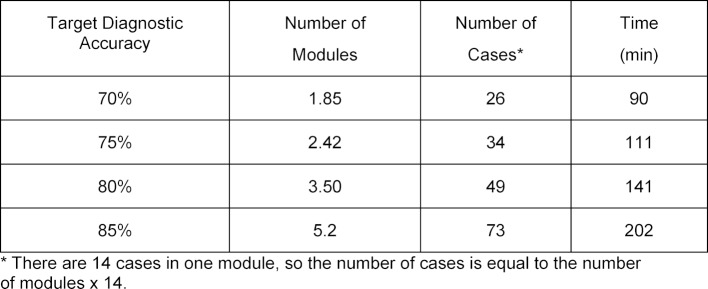
Number of Modules Completed and Total Practice Time required to achieve pre-defined Target Diagnostic Accuracies.
