# Supplementary material for: Quantifying the medical student learning curve for ECG rhythm strip interpretation using deliberate practice
Source: GMS J Med Educ. 2019 Aug 15;36(4):Doc40. doi: 10.3205/zma001248 (PMC6737266; doi:10.3205/zma001248)
Supplement: Statistical analysis of practice data [file JME-36-4-40-s-001.pdf]

Statistical test	ANCOVA*	paired t test, Bonferroni corrected	ANCOVA*	paired t test, Bonferroni corrected
Comparitors	School 1 vs. School 2	Compared to prior module	School 1 vs. School 2	Compared to prior module
Metric	Diagnostic accuracy	Diagnostic accuracy	Time per module	Time per module
Module 1	*t*(299) = XX, p = 0.63	No prior module	*t*(299) = XX, p = 0.19	No prior module
Module 2	*F*(1, 278) = 1.68, *p* = 0.20	*t*(284) = -8.03, p < 0.001	*F*(1, 278) = 0.44, *p* = 0.51	*t*(284) = 10.16, p < 0.001
Module 3	*F*(1, 241) = 2.40, *p* = 0.12	*t*(246) = -6.84 p < 0.001	*F*(1, 241) = 5.19, p = 0.024	*t*(246) = 7.82 p < 0.001
Module 4	*F*(1, 169) = 0.00, *p* = 0.97	*t*(174) = -2.21, p = 0.07	*F*(1, 169) = .044, p = 0.83	*t*(174) = 3.95, p = 0.007
Module 5	*F*(1, 112) = 3.13, *p* < 1.64	*t*(117) = -2.65, p = 0.02	*F*(1, 112) = 2.87, *p* = 0.09	*t*(117) = 4.39 p < 0.001
Module 6	n/a	n/a	*F*(1, 77) = 0.52, *p* = 0.47	*t*(81) = 3.25, p = 0.006
Module 7	n/a	n/a	*F*(1, 50) = 3.55, *p* = 0.07	*t*(54) = 3.16, p = 0.009
Module 8	n/a	n/a	*F*(1, 33) = 0.01, *p* = 0.91	*t*(37) = 4.33, p < 0.001

*School 1 vs. School 2 comparisons in Module 1 were assessed by t-test because no comparison to prior module was possible.
n/a = not assessed. Decision to not assess was made *a priori* based on visual inspection of the Gompertz and Michaelis-Mentin curves.
