# Supplementary material for: Quantifying the medical student learning curve for ECG rhythm strip interpretation using deliberate practice
Source: GMS J Med Educ. 2019 Aug 15;36(4):Doc40. doi: 10.3205/zma001248 (PMC6737266; doi:10.3205/zma001248)
Supplement: Pre-survey completed by 298 students [file JME-36-4-40-s-002.pdf]

PRE-SURVEY COMPLETED BY 298 STUDENTS

[RESPONSES IN BRACKETS]

1. What is your gender?
   a. Male [46%]
   b. Female [54%]
2. What is your age? (type number only please) [24.9 (4.2) years]
3. How much time practicing ECG interpretation have you done previously?
   a. None [43%]
   b. 1-30 minutes [19%]
   c. 31-60 minutes [14%]
   d. 60-120 minutes [13%]
   e. >120 minutes [11%]
4. How confident are you at reading ECG's?
   a. Not at all (I would be completely guessing) [20%]
   b. Very low (I might get 1 right) [28%]
   c. Low (I might get a couple right) [37%]
   d. High (I should get most right) [13%]
   e. Very high (I expect to get nearly all right) [2%]
5. Have you studied using online practice modules before for medical skills?
   a. Never [60%]
   b. Yes, but not for ECG [35%]
   c. Yes, and it was for ECG [5%]
6. Do you think practicing is required for learn ECG interpretation?
   a. Not required [0%]
   b. Probably not needed [0%]
   c. I don't know [1%]
   d. Probably important [11%]
   e. Absolutely required [88%]

7. Do you expect online practice modules for ECG will be EFFICIENT (good learning for your time spent)?
    a. Not at all [0%]
    b. Probably not [0%]
    c. Not sure [7%]
    d. Probably yes [38%]
    e. For sure yes [55%]
8. Do you expect online practice modules for ECG will be EFFECTIVE (they will actually work for you)?
    a. Not at all [0%]
    b. Probably not [1%]
    c. I don't know [6%]
    d. Probably effective [62%]
    e. Extremely effective [31%]
9. If you have any comments, please type below:

POST SURVEY COMPLETED BY 174 STUDENTS

1. How confident are you at reading ECG's?
    a. Not at all (I would be completely guessing) [0%]
    b. Very low (I might get 1 right) [1%]
    c. Low (I might get a couple right) [13%]
    d. High (I should get most right) [75%]
    e. Very high (I expect to get nearly all right) [11%]
2. Do you think practicing is required for learn ECG interpretation?
    a. Not required [0%]
    b. Probably not needed [0%]
    c. I don't know [1%]
    d. Probably important [4%]
    e. Absolutely required [95%]

3. Do you think online practice modules for ECG is EFFICIENT (good learning for your time spent)?
    a. Not at all [0.5%]
    b. Probably not [0.5%]
    c. Not sure [1%]
    d. Probably yes [24%]
    e. For sure yes [74%]
4. Do you expect online practice modules for ECG will be EFFECTIVE (they will actually work for you)?
    a. Not at all [1%]
    b. Probably not [0%]
    c. I don't know [3%]
    d. Probably effective [37%]
    e. Extremely effective [60%]
5. How important was the instant feedback to you?
    a. Not at all. It did not help [0%]
    b. Not bad. Was occasionally useful [2%]
    c. I don't know [2%]
    d. Good. It helped a fair bit. [31%]
    e. Excellent. Is absolutely required. [65%]
6. Did you have fun working on the practice modules?
    a. Not at all. They were painful [1%]
    b. Not really, but they were not painful [4%]
    c. I don't know [3%]
    d. They were a little enjoyable [48%]
    e. They were very fun [44%]

7. Would you want this format of practice for other skills (x-rays or ultrasound for example)?

    a. Not at all [1%]

    b. Not really [1%]

    c. I don't know [0%]

    d. Probably yes [22%]

    e. For sure yes [76%]

8. If you have any comments, please type below:
